# Transcription Expression and Clinical Significance of Dishevelled-3 mRNA and **δ**-Catenin mRNA in Pleural Effusions from Patients with Lung Cancer

**DOI:** 10.1155/2012/904946

**Published:** 2012-02-23

**Authors:** Xiao-Yan Li, Shu-Li Liu, Na Cha, Yu-Jie Zhao, Shao-Cheng Wang, Wei-Nan Li, En-Hua Wang, Guang-Ping Wu

**Affiliations:** ^1^Department of Pathology, The First Affiliated Hospital and College of Basic Medical Sciences, China Medical University, Shenyang 110001, China; ^2^Center of Biochip, College of Basic Medical Sciences, China Medical University, Shenyang 110001, China

## Abstract

*Objective*. To evaluate diagnostic utility of Dishevelled-3 (DVL-3) mRNA and **δ**-catenin mRNA expression in pleural effusions of patients with lung cancer. *Methods*. DVL-3 mRNA and **δ**-catenin mRNA levels were assessed by performing RT-PCR on pleural effusion specimens from patients with lung cancer (*n* = 75) and with lung benign disease (*n* = 51). *Results*. The expressions of DVL-3 mRNA and **δ**-catenin mRNA were significantly higher in malignant than in benign lung disease (*P* < 0.01) and were obviously higher than cytology in adenocarcinoma (*P* < 0.01). In single use, DVL-3 mRNA had the highest specificity (94.1%) and PPV (95.7%), whereas **δ**-catenin mRNA had the highest sensitivity (92.0%) and NPV (88.5%). When combinations of markers were evaluated together, DVL-3 mRNA and **δ**-catenin mRNA gave a high-diagnostic performance: sensitivity of 100.0%, NPV of 100.0%, and accuracy of 96.0%, respectively. *Conclusion*. As molecular markers of detecting pleural micrometastasis, DVL-3 mRNA and **δ**-catenin mRNA are helpful to diagnose the cancer cells in pleural effusions of patients with lung cancer.

## 1. Introduction

Pleural effusion is a common clinical complication produced by a wide variety of diseases. Approximately 20% of pleural effusions are due to malignancy, and 50% of these are due to primary lung cancer [1]. A malignant pleural effusion may be the initial presentation of cancer in 10 to 50% of patients [2]. The cytologic diagnosis of pleural effusions can be difficult and usually detect only 50–60% of malignant pleural effusions [3], especially in specimens containing abundant reactive mesothelial cells. Distinguishing carcinoma cells from reactive mesothelial cells in such fluid are particularly challenging when there are relatively few carcinoma cells [4]. Additional techniques, such as immunocytochemistry and enzyme-linked immunosorbent assay, provide significant help in this differential diagnosis [5, 6]. So far, many tumor markers directed against specific cell type antigens have been used in pleural effusions to enhance the cytological diagnosis, with varying degrees of efficacy [7, 8], but the optimum panel of tumor markers still has to be reported. Moreover, the definition of earlier diagnosis of pleural effusions of patients with lung cancer by means of detecting tumor marker mRNA in occult lung cancer cells had previously been reported in our studies [9, 10].

 DVL-3 belongs to Dishevelled (Dvl) family proteins which are cytoplasmic mediators of the Wnt/beta-catenin signaling pathway and have recently been proved to be overexpressed in nonsmall cell lung cancer (NSCLC), especially in adenocarcinomas [11]. **δ**-catenin is a component of the synaptic adherens junction that is necessary for normal learning and memory [12]. As a member of the p120 catenin (p120ctn) subfamily, **δ**-catenin is the only one that its primary expression is restricted to the brain. However, in recent years **δ**-catenin has shown overexpression in human lung cancer and has proved a useful marker in differentiating a malignant from a benign origin, the effects of **δ**-catenin expression in lung cancer still need to be clarified [13, 14].

 The main purpose of this study was to determine the diagnostic capacity in pleural effusions of tumor markers DVL-3 mRNA and **δ**-catenin mRNA. In particular, we evaluate the value of DVL-3 mRNA and **δ**-catenin mRNA in differentiating the pleural effusions of patients with lung cancer from those of patients with benign lung disease.

## 2. Materials and Methods

### 2.1. Patients

The study was conducted in accordance with the regulations of the institutional review boards at China Medical University and was performed at The First Affiliated Hospital, China Medical University. Internal review board approval for this study and/or the informed consent of the patients was obtained. A total of 126 pleural effusion samples collected from the patients at the Laboratory of Cytopathology of the First Affiliated Hospital of China Medical University from May 2010 to June 2011 were included in this study. There were 68 males (54%) and 58 females (46%). Samples consisted of 75 malignant effusions and 51 benign effusions. The effusions were classified as benign or malignant on the basis of their definite pathologic diagnosis.

A total of 51 effusions were defined as benign including parapneumonic (26) and tuberculosis (25) and 75 as adenocarcinoma. Of the 51 patients with benign effusion, 38 were men (74.5%) and 13 were women (25.5%), with a mean age of 61.4 years (range, 20–84). Of the 75 patients with malignant effusion, 30 were men (40.0%) and 45 were women (60.0%), with a mean age of 60.1 years (range, 29–80).

The effusions were considered malignant if malignant cells were found on cytologic examination or in a biopsy specimen. Only specimens diagnosed as primary malignancies of lung or pleura were considered; malignancies of any other cause were excluded.

Tuberculous pleurisy was diagnosed if one of the following criteria was met: identification of bacillus in pleural fluid or biopsy specimen cultures; the presence of caseous granulomas in pleural biopsy tissue; radiological and clinical evidence of tuberculous pleurisy with acid-fast bacilli-positive sputum, followed by response to antituburculous therapy.

Parapneumonic pleurisy was determined when there was an acute febrile illness with purulent sputum, pulmonary infiltrate, and responsiveness to antibiotic treatment or identification of the microorganism in the pleural effusion in the absence of any other cause associated with pleural effusions.

### 2.2. Preparation of Cells from Pleural Effusions

All specimens were received as fresh effusion, with a volume range of 20–2,000 mL. The specimens were centrifuged for 30 minutes at 2,000 rpm at 4°C. The resulting pellet was used for the preparation of two cytological smears (alcohol fixed, Papanicolau stained), and the rest of the pellet was stored at −70°C until being used for RNA extraction.

### 2.3. RT-PCR Analysis

*Total RNA Extraction. *Total RNA was extracted from the cell pellet using Trizol Reagent (Gibco, Life Technologies; Rockville, MD) according to the manufacturer's instruction. Then, total RNA was diluted in RNase-free water and quantified by measurement of absorbance at 260 and 280 nm. The range of OD260/OD280 of extraction of total RNA from all the specimens is 1.82–2.01.

**Figure 1:**
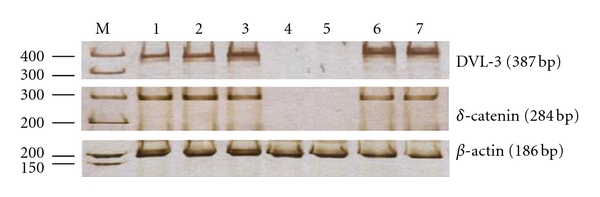
RT-PCR amplified products of DVL-3, **δ**-catenin, and **β**-actin in pleural effusions of patients with lung cancer and benign diseases. The 387, 284, and 186 bp DNA fragments were expected to be amplified from DVL-3, **δ**-catenin, and **β**-actin cDNAs, respectively. Lane M represented DNA marker (50–500 bp). Lanes 1, 2, 3, 6 and 7 represented pleural effusion of patients with lung cancer, respectively. Lanes 4 and 5 represented pleural effusion of patients with benign diseases, respectively.

 Reverse transcription was done with the TaKaRa RNA PCR KIT (AMV) Ver.3.0 (TaKaRa; Lianxing Bio, Dalian, China). Following the manufacture's instructions, total RNA (0.5 ug) was converted to first-strand cDNA in 10-uL reaction mixture, which contains 1 uL 0.5 g/l total RNA, 2 uL 25 mmol/L MgCl_2_, 1 uL 10 × RT buffer, 1 uL 10 × dNTP mixture (10 mM each), 0.25 uL RNase inhibitor, 0.5 uL 5 U/uL AMV (Avain Myeloblastosis Virus) Reverse Transcriptase, 0.5 uL. 0.5 g/l Oligo dT-Adaptor Primer, and 3.75 uL RNase-free dH_2_O, at 42°C for 30 minutes, 99°C for 3 minutes, and 5°C for 5 minutes. PCR was done in 50-ul reaction mixture containing 4-uL cDNA, 31.7-uL ddH2O, 10 uL 5 × PCR buffer, 4 uL for both sense and antisense primers of DVL-3, **δ**-catenin, and **β**-actin, 0.3 uL 5 U/uL TaKaRa EX Taq HS. Primers of **β**-actin were used to check RNA intergrity and the efficiency of the reverse transcription step. The primers sequences, melting temperature, and expected length of synthesis fragments were shown in Table 1. After a denaturing step at 94°C for 2 minutes, PCR was done at 94°C for 30 seconds, at 57°C for 40 seconds, and at 72°C for 40 seconds for 35 cycles. PCR products were separated by electrophoresis on 8% polyacrylamide gels, followed by staining with AgNO_3_, as shown in Figure 1. The whole-test analysis was performed without any knowledge of the patients' clinical findings.

### 2.4. Quality Control

The nucleotide sequence of PCR product was subcloned and sequenced by dideoxy chain termination and confirmed to be 100% homologous to human DVL-3 and **δ**-catenin. A GenBank database search verified that the sequences are specific to DVL-3 and **δ**-catenin.

### 2.5. Statistical Analysis

Statistical analysis was performed using the *χ* ^2^ test or Fisher's exact test when theoretical effectiveness was insufficient. The level of statistical significance was set at *P* < 0.05. The utility of each marker was determined by means of sensitivity, specificity, PPV, NPV, and accuracy. Diagnostic performance of the combination of DVL-3 mRNA and **δ**-catenin mRNA was calculated in parallel text. Calculations for combinations of DVL-3 mRNA and **δ**-catenin mRNA were assessed as “positive” if RT-PCR result was seen with either or both positive results and “negative” if both were negative.

## 3. Results

The results of DVL-3 mRNA expression, **δ**-catenin mRNA expression, and cytological assessment of pleural effusion specimens from patients with adenocarcinoma, parapneumonic, and tuberculosis are presented in Table 2. Both DVL-3 mRNA and **δ**-catenin mRNA were significantly more likely to be expressed in malignant than in benign lung disease (*P* < 0.01), and the expressions of DVL-3 mRNA and **δ**-catenin mRNA were more likely to be positive than cytology in adenocarcinoma (*P* < 0.01). Moreover, the expression of DVL-3 mRNA was positively correlated with **δ**-catenin mRNA (*r* = 0.743).

Table 3 shows the results obtained by cytological assessment as compared with those obtained by RT-PCR or histology, for pleural effusions from patients with benign lung disease and with adenocarcinoma. There were significantly fewer false negative results by RT-PCR than by cytology because RT-PCR facilitates the detection of few or single carcinoma cells in pleural effusion specimens. Twenty-seven and thirty specimens gave false-negative results by cytology but positive results by detecting DVL-3 mRNA and **δ**-catenin mRNA, respectively; these were fifteen specimens with suspected cancer cells and twelve specimens with reactive mesothelial cells by detecting DVL-3 mRNA, and eighteen specimens with suspected cancer cells and twelve with reactive mesothelial cells by detecting **δ**-catenin mRNA, respectively. There were two specimens with pneumonia and one specimen positive for tuberculosis that gave false-positive results by detecting DVL-3 mRNA, and four specimens with pneumonia and one specimen positive for tuberculosis that gave false-positive results by detecting **δ**-catenin mRNA, respectively.

Table 4 shows the sensitivity, specificity, PPV, NPV, and accuracy of DVL-3 mRNA, **δ**-catenin mRNA, combinations of DVL-3 mRNA and **δ**-catenin mRNA, and cytology, with respect to the histological diagnosis of lung cancer. In single, DVL-3 mRNA had the highest specificity (94.1%) and PPV (95.7%), whereas **δ**-catenin mRNA had the highest sensitivity (92.0%) and NPV (88.5%). When combinations of DVL-3 mRNA and **δ**-catenin mRNA were evaluated together, DVL-3 mRNA and **δ**-catenin mRNA gave a high- diagnostic performance: sensitivity of 100.0%, NPV of 100.0%, and accuracy of 96.0%, respectively. These were evidently higher than in single use and cytology. Although specificity (90.2%) and PPV (93.8%) were lower for combinations of DVL-3 mRNA and *δ*-catenin mRNA than for cytology (100%), there were no statistical significant in these differences (*P* > 0.05).

## 4. Discussion

Morphological differentiation of reactive mesothelial cells from carcinoma cells in pleural effusions can be a diagnostic challenge. Adenocarcinoma metastatic to the pleural membrane is often associated with prominent mesothelial hyperplasia and may result in diagnostic confusion. The difficulty is obviously greater when neoplastic cells show only slight atypia or when they are scarce in the effusion [7]. False-negative results of cytological examination of pleural fluid are a serious problem. Such errors in diagnosis usually are caused by misinterpretation of adenocarcinoma cells as reactive mesothelial cells [4]. The rate of false-positive diagnoses also is significant and often caused by overinterpretation of reactive mesothelial cells as malignant cells [15]. Immunocytochemistry can greatly aid in such diagnostic dilemmas, but, currently, available markers have varying sensitivities and specificities for mesothelial cell or cells of epithelial differentiation [16, 17]. Recently, we have evaluated RT-PCR techniques for the detection of cancer cells in pleural effusions of patients with lung cancer and have demonstrated that these techniques are more sensitive than immunocytochemistry [9, 10].

DVL-3 is a member of the human dishevelled family, located on chromosome 3q27 [18], is abnormally expressed in nonsmall cell lung cancer, and affect lung cancer cell invasiveness and metastasis [11]. **δ**-catenin belongs to the p120-catenin protein family, located on chromosome 5p15.2 [19] and contains a ten-armadillo repeat domain,which has 47% similarity to that of p120ctn [20]. The mRNA and protein expression of **δ**-catenin was increased in lung cancer tissues and its positive expression rate was significantly increased in adenocarcinoma, stages III-IV, paired lymph node metastasis lesions, and primary tumours with lymph node metastasis [13], but little is known about the positive expression rate in pleural effusions from patients with lung cancer and the correlation between DVL-3 and **δ**-catenin.

Identification of molecular markers for disease progression would be of great clinical value. RT-PCR is a sensitive method that can objectively detect even one cancer cell among 10^6^ cells [21, 22]. In the present study, the expression frequencies of DVL-3 mRNA and **δ**-catenin mRNA were significantly greater in the adenocarcinoma group (*P* < 0.01) compared with the benign lung disease group. The rate of positive diagnosis was significantly greater by RT-PCR than by cytology in the adenocarcinoma group. Moreover, the expression of DVL-3 mRNA was positively correlated with **δ**-catenin mRNA (*r* = 0.743). Twenty-seven and thirty specimens gave false-negative results by cytology but positive results by detecting DVL-3 mRNA and **δ**-catenin mRNA, respectively; these were fifteen specimens with suspected cancer cells and twelve specimens with reactive mesothelial cells by detecting DVL-3 mRNA, and eighteen specimens with suspected cancer cells and twelve with reactive mesothelial cells by detecting **δ**-catenin mRNA, respectively. False-negative results on cytological examination may be due to cells that are only slightly atypical or to a scarcity of cells in pleural effusions of patients with lung cancer. Although all eighteen specimens with suspicious cytology contained malignant cells and were positive by detecting **δ**-catenin mRNA, atypical cytology was not necessarily associated with malignancy, even when **δ**-catenin mRNA was positive. Three and five specimens gave false-positive results by detecting DVL-3 mRNA and *δ*-catenin mRNA, respectively, giving a PPV of 95.7% and 93.2%, as compared with 100% by cytology. Therefore, DVL-3 mRNA and **δ**-catenin mRNA are sensitive molecular markers for the diagnosis of lung cancer.

At present, there is an obvious limitation in the international TNM staging of nonsmall cell lung carcinoma that was absent from the interpretation of occult tumor cells [23, 24]. Additionally, the inclusion of the interpretation of occult micrometastases in the current TNM staging system is also recommended by the International Union Against Cancer [25]. Thus, detecting DVL-3 mRNA and **δ**-catenin mRNA in a pleural effusion can provide a supplement to TNM staging.

In conclusion, RT-PCR detection of DVL-3 mRNA and *δ*-catenin mRNA showed better diagnostic performance compared with cytology, especially when combinations of DVL-3 mRNA and **δ**-catenin mRNA were evaluated together with a sensitivity of 100% and an NPV of 100%. These findings suggest that detection of DVL-3 mRNA and **δ**-catenin mRNA in pleural effusions from patients with lung cancer could possibly be used as ancillary tools for the diagnosis of lung cancer. This sensitive, accurate, noninvasive method may be helpful as a complementary tool that will facilitate the establishment of a diagnosis of lung cancer.

## Figures and Tables

**Table 1:** Sequences and features of the primers used for RT-PCR.

Names	Sequence of sense and antisense of primers	Melting temperature	Length
DVL-3	5′- AACCAGGGGGTTATGATAGCTC-3′ 5′- TATCTCCTGGCTCGATGCGTCC- 3′	57°C	387 bp
*δ*-catenin	5′-TACTCCGCAAGACGACTGACC-3′ 5′-CCATCACACTCTCTCATCCTTCTG-3′	57°C	284 bp
*β*-actin	5′-TGGCACCCAGCACAATGAA −3′ 5′-CTAAGTCATAGTCCGCCTAGAAGCA −3′	55°C	186 bp

bp: base pairs.

**Table 2:** Results for DVL-3 mRNA, **δ**-catenin mRNA expression by RT-PCR and for cytological assessment in pleural effusions of patients with benign or malignant lung lesions.

Group	*n*	DVL-3 mRNA		*δ*-catenin mRNA		Cytology	
		+	−	+	−	+	−
Pneumonia	26	2	24	4	22	0	26
Tuberculosis	25	1	24	1	24	0	25
Adenocarcinoma	75	67^∗§^	8	69^∗§^	6	44	31

*P < 0.01 as compared to pneumonia and tuberculosis. ^§^ *P* < 0.01 as compared to cytology. (i) The DVL-3 mRNAs were positively correlated with **δ**-catenin mRNA (*r* = 0.743). Date are number of specimens.

**Table 3:** Results obtained by cytological assessment as compared with those obtained by RT-PCR for DVL-3 mRNA and **δ**-catenin mRNA or by histology in pleural effusions specimens from patients with benign or malignant lung lesions.

Cytology	Total	DVL-3 mRNA		*δ*-catenin mRNA		Histology	
	*n*	+	−	+	−	+	−
Pneumonia	40	2	38	4	36	0	40
Tuberculosis	11	1	10	1	10	0	11
RMC	13	12	1	12	1	13	0
Suspected to be malignant	18	15	3	18	0	18	0
Adenocarcinoma cells	44	40	4	39	5	44	0

Total	126	70	56	74	52	75	51

Date are number of specimens. RMC: reactive mesothelial cells.

**Table 4:** Accuracy of RT-PCR for detection of DVL-3 mRNA, *δ*-catenin mRNA and combination of them compared with cytology for the diagnosis of lung cancer.

Variables	Single		Combination of	Cytology
	DVL-3	**δ**-catenin	DVL-3 plus *δ*-catenin	
Sensitivity (%)	89.3	92.0	100.0*	58.7
Specificity (%)	94.1	90.2	90.2	100.0
Accuracy (%)	91.3	91.3	96.0*	75.4
PPV (%)	95.7	93.2	93.8	100.0
NPV (%)	85.7	88.5	100.0*	62.2

PPV: positive predictive value; NPV: negative predictive value; *P < 0.01 comparing RT-PCR of combination group with cytology.
